# Protected risk stratification with the wearable cardioverter-defibrillator: results from the WEARIT-II-EUROPE registry

**DOI:** 10.1007/s00392-020-01657-2

**Published:** 2020-05-06

**Authors:** Christian Veltmann, Stefan Winter, David Duncker, Carsten G. Jungbauer, Nadine K. Wäßnig, J. Christoph Geller, Julia W. Erath, Olaf Goeing, Christian Perings, Michael Ulbrich, Mattias Roser, Daniela Husser, Laura S. Gansera, Korkut Soezener, Frank Michael Malur, Michael Block, Thomas Fetsch, Valentina Kutyifa, Helmut U. Klein

**Affiliations:** 1 Rhythmology and Electrophysiology, Department of Cardiology and Angiology, Hannover Medical School, Carl-Neuberg-Str. 1, 30625 Hannover, Germany; 2 St. Vinzenz-Hospital Köln, Cologne, Germany; 3 University Hospital Regensburg, Regensburg, Germany; 4 Universitätsklinikum Dresden, Herzzentrum, Dresden, Germany; 5 Arrhythmia Section, Division of Cardiology, Zentralklinik Bad Berka, Bad Berka, Germany; 6 Otto-Von-Guericke University School of Medicine, Magdeburg, Germany; 7 Abteilung für Klinische Elektrophysiologie, Medizinische Klinik III, Universitätsklinikum Frankfurt, Frankfurt, Germany; 8 Sana Klinikum Lichtenberg, Berlin, Germany; 9 St.-Marien Hospital Lünen, Lünen, Germany; 10 Klinikum Siegburg, Siegburg, Germany; 11 Klinikum Benjamin Franklin, Charité Berlin, Berlin, Germany; 12 Klinik für Kardiologie, Herzzentrum Leipzig, Leipzig, Germany; 13 Klinik für Kardiologie, Klinikum Augsburg, Augsburg, Germany; 14 Klinikum Frankfurt-Hoechst, Frankfurt, Germany; 15 Helios Klinikum Erfurt, Erfurt, Germany; 16 Klinik für Kardiologie, Klinikum Augustinum München, Munich, Germany; 17 CRI-Clinical Research Institute München, Munich, Germany; 18 Medical Center, Clinical Cardiovascular Research Center, University of Rochester, Rochester, NY USA

**Keywords:** Heart failure, Sudden cardiac death, Implantable cardioverter-defibrillator, Wearable cardioverter-defibrillator

## Abstract

**Background:**

The prospective WEARIT-II-EUROPE registry aimed to assess the value of the wearable cardioverter-defibrillator (WCD) prior to potential ICD implantation in patients with heart failure and reduced ejection fraction considered at risk of sudden arrhythmic death.

**Methods and results:**

781 patients (77% men; mean age 59.3 ± 13.4 years) with heart failure and reduced left ventricular ejection fraction (LVEF) were consecutively enrolled. All patients received a WCD. Follow-up time for all patients was 12 months. Mean baseline LVEF was 26.9%. Mean WCD wearing time was 75 ± 47.7 days, mean daily WCD use 20.3 ± 4.6 h. WCD shocks terminated 13 VT/VF events in ten patients (1.3%). Two patients died during WCD prescription of non-arrhythmic cause. Mean LVEF increased from 26.9 to 36.3% at the end of WCD prescription (*p* < 0.01). After WCD use, ICDs were implanted in only 289 patients (37%). Forty patients (5.1%) died during follow-up. Five patients (1.7%) died with ICDs implanted, 33 patients (7%) had no ICD (no information on ICD in two patients). The majority of patients (75%) with the follow-up of 12 months after WCD prescription died from heart failure (15 patients) and non-cardiac death (15 patients). Only three patients (7%) died suddenly. In seven patients, the cause of death remained unknown.

**Conclusions:**

Mortality after WCD prescription was mainly driven by heart failure and non-cardiovascular death. In patients with HFrEF and a potential risk of sudden arrhythmic death, WCD protected observation of LVEF progression and appraisal of competing risks of potential non-arrhythmic death may enable improved selection for beneficial ICD implantation.

**Graphic abstract:**

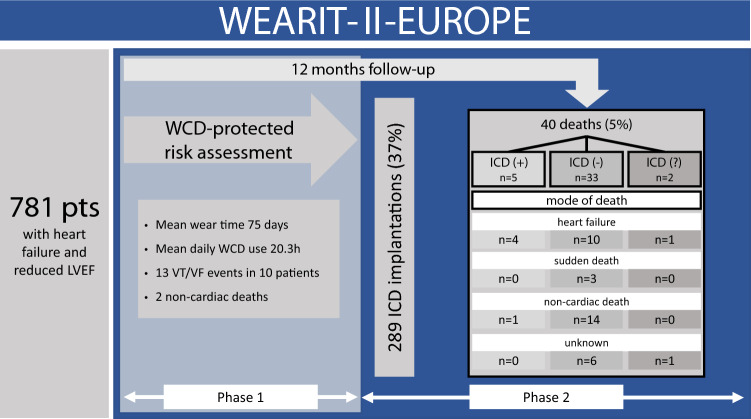

## Introduction

Numerous randomized trials have proven the benefit of primary prevention cardioverter-defibrillator (ICD) implantation. Currently, assumption of a life-threatening arrhythmic risk is almost exclusively based on reduced left ventricular ejection fraction (LVEF ≤ 35%) and clinical symptoms of heart failure [1]. Conditions or causes of an increased arrhythmic risk may change over time, and the threat of an arrhythmic death may lower or no longer exists [2]. Although the wearable cardioverter-defibrillator (WCD) has been introduced into clinical practice more than 15 years ago, general acceptance of this device for risk assessment is still limited [3–8]. Use of the WCD seems to be a suitable approach to perform protected risk assessment until the decision to either implant or withhold an ICD for primary prevention. Recently, the WEARIT-II-US registry has demonstrated that life-threatening arrhythmic events during the time of WCD wearing can reliably be terminated by shock delivery [9]. In contrast to WEARIT-II-US and other previous registries, the objective of the current WEARIT-II-EUROPE registry was to demonstrate the clinical value of the WCD prescription in patients with heart failure and reduced ejection fraction (HFrEF) prior to deciding for or against primary preventive ICD implantation. During the time of WCD prescription, physicians had the opportunity to monitor patients’ ECG, to assess the improvement of cardiac function and to appraise patients’ overall mortality risk and particularly the likelihood of arrhythmic death. The current registry uniquely included a 1-year follow-up after the WCD prescription period to analyze the clinical outcome and mode of death.

To increase the benefit of primary prevention ICD therapy, we hypothesize that protected risk assessment prior to potential ICD implantation can effectively be performed under the umbrella of the WCD.

## Methods

The WEARIT-II-EUROPE registry is a prospective, multicenter, observational registry of patients to whom the WCD was prescribed in 30 German tertiary clinical centers during a time period of 21 months (January 2014–September 2015). All enrolled patients had a potential risk of life-threatening ventricular arrhythmias. Prescription of the WCD, its wearing time and therapy thereafter were left to the discretion of the attending physicians.

The registry consisted of two phases: Phase 1 of the registry started after patients’ signed consent with prescription of the WCD and ended with termination of WCD prescription. Phase 2 started after WCD prescription up to a follow-up of 12 months after WCD prescription. At WCD prescription, patients were categorized into specific groups of pre-specified WCD indications: (1) newly diagnosed non-ischemic cardiomyopathy (NICM) with reduced LV function (LVEF ≤ 35%) including myocarditis, cardiac sarcoidosis, peripartum cardiomyopathy, or idiopathic cardiomyopathy; (2) new hospitalization for acute heart failure (HF) with structural heart disease regardless of the underlying etiology; (3) severely reduced LVEF (≤ 35%) within the first week following acute myocardial infarction (AMI), independent of performed revascularization (PCI) or early occurring VT/VF events; (4) reduced LV function (LVEF ≤ 35%) less than 1 month after elective PCI procedures or recent coronary artery bypass grafting (CABG); and (5) other risk assessment indications with or without structural heart disease in the presence of non-sustained ventricular tachycardia, ECG abnormalities, unexplained syncope or aborted cardiac arrest of unknown cause, including patients with presumed inherited arrhythmia disorders.

During Phase 2 of the registry, all major clinical events, LVEF re-assessment, ICD implantation, shock delivery, and ICD complications were recorded.

The main objectives of the registry were to investigate the clinical course after prescription of the WCD with respect to improvement of left ventricular function (LVEF) under guideline-directed medical therapy (GDMT) and overall mortality at 12 months after WCD prescription.

All clinical data and events were stored in an electronic CRF system. The registry protocol was approved by the Central Ethical Committee Board (IRB) of the State of Bavaria, Germany, and the ethical committee boards of each participating center. All patients gave written informed consent.

### Arrhythmic events during WCD prescription

Centers had continuous access to patients’ ECG and WCD wearing compliance via telemonitoring (LifeVest Network^®^, ZOLL-Inc. Pittsburgh, USA). Recorded WCD events were classified as true arrhythmic events or artifacts. Any arrhythmia episode, separated by at least 30 min from a previous episode, was considered an independent arrhythmic event. Arrhythmic events were classified as either potentially life-threatening or non-life-threatening events. Life-threatening episodes were ventricular fibrillation (VF), and sustained ventricular tachycardia (VT) (> 30 s). Non-life-threatening arrhythmia episodes were non-sustained VT, paroxysmal or persistent atrial fibrillation, atrial flutter, or any regular supraventricular tachycardia (SVT). Separately, severe bradycardia (< 20 bpm) or asystole were adjudicated as bradycardia events. WCD shocks delivered for non-life-threatening events were considered as inappropriate. Use of WCD response buttons for arrhythmic events was also analyzed. WCD data and all arrhythmic events were blindly reviewed by one of the authors (HUK).

### Statistical analysis

Continuous variables are reported as mean/median with interquartile ranges. Categorical data are reported as frequencies and percentages. Groups were compared using Student’s *t* test for testing differences in continuous variable LVEF points from different time points: inclusion, Phase 1 and Phase 2 and Pearson’s chi-square test for testing the independency of categorical variables LV-EF group/NYHA and the clinical phase as appropriate. The cumulative incidence of arrhythmic events and time to first life-threatening VT/VF event are displayed using the Kaplan–Meier method. Similarly, cumulative survival with or without ICD implantation was analyzed. Cox proportional hazards regression models were assessed on age groups, LVEF categories, different WCD indications and a stepwise variable selection was performed on the models. Statistical tests were performed 2-sided and nominal *p* values of < 0.05 were considered statistically significant. Analyses were performed with R project for statistical computing (version 3.0.2 or later).

## Results

### Patient population

**Fig. 1:**
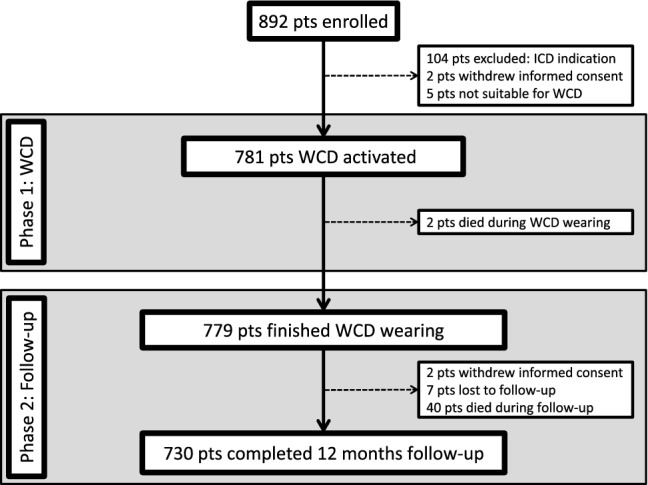
Flow chart of the WEARIT-II-EUROPE registry

The WEARIT-II-EUROPE registry enrolled 892 patients; two patients withdrew permission to use their personal data prior to WCD activation, and five patients were considered not suitable for WCD wearing. Therefore, the registry population consisted of 885 patients (Fig. 1). A total of 104 patients were excluded from this current analysis since they already had an established ICD indication. In 63 patients, the WCD was prescribed after ICD removal and in 41 patients ICD implantation was postponed due to comorbidities.

The remaining 781 patients without already confirmed ICD indication were analyzed. Patient baseline characteristics and indications for WCD prescription are shown in Table 1. 599 men (77%) and 182 (23%) women were enrolled with a mean age of 59.3 ± 13.4 years. Patients were categorized according to the WCD prescription. In 249 patients (32%), the WCD was prescribed because of newly diagnosed non-ischemic cardiomyopathy; in 230 patients (30%) hospitalized with newly diagnosed acute heart failure; in 128 patients (16%) immediately (< 1 week) after acute myocardial infarction with LVEF ≤ 35%; in 72 patients (9%) who underwent revascularization procedures without concomitant acute myocardial infarction; and in 102 patients (13%) for various reasons (unknown causes of syncope, aborted cardiac arrest or presumed inherited arrhythmia syndromes).

**Table 1:** Baseline characteristics

	781 patients
Mean age (years)	59.3 ± 13.4
Males	599 (77%)
Mean BMI (kg/m^2^)	28.2 ± 5.3
Mean LVEF (%)	26.9 ± 10.3
LVEF ≤ 35%	700 (90%)
LVEF > 35%	81 (10%)
WCD indications	
Non-ischemic cardiomyopathy	249 (32%)
Recent onset/impairment of heart failure	230 (30%)
Acute myocardial infarction	128 (16%)
Revascularization procedures (PCI/CABG)	72 (9%)
Other risk stratification	102 (13%)
WCD wearing time	
Mean wearing time	75.0 ± 47.7 days
Mean WCD wearing per day	20.3 ± 4.6 h/day

*BMI* Body mass index, *CABG* Coronary artery bypass grafting, *LVEF* Left ventricular ejection fraction, *PCI* Percutaneous coronary intervention, *WCD* Wearable cardioverter-defibrillator

#### Phase I: WCD prescription period

Mean WCD prescription time of the total patient cohort was 75.0 ± 47.7 days (interquartile range 45–93 days); while, the mean daily usage was 20.3 ± 4.6 h (interquartile range 19.5–23.2 h). Arrhythmic events during WCD prescription are shown in Table 2. Ten patients (1.3%) received appropriate shocks for 13 VT/VF episodes. Every sustained VT/VF event was terminated with a single WCD shock. Two patients (0.3%) received a single inappropriate shock for rapid atrial fibrillation. Twenty-two patients pressed the response buttons of the WCD to withhold shock delivery for a total of 47 tachycardia episodes (24 sustained VTs in 12 patients, and 23 atrial tachyarrhythmias in 10 patients). Distribution of VT/VF events within the various WCD indications is listed in Table 2. No patient had untreatable or undetected ventricular arrhythmic events. No bradycardia events (< 20 bpm) occurred. During WCD prescription, two patients died 17 and 43 days after WCD prescription. One patient died from terminal heart failure, the other patient from non-cardiac cause (renal failure).

**Table 2:** Arrhythmic events during WCD wearing

	Patients (*n*)	Events (*n*)
Total	781	83
WCD shocks	12 (1.5%)	15
Appropriate shocks	10 (1.3%)	13
Inappropriate shocks	2 (0.3%)	2
Ventricular tachyarrhythmias	21 (2.7%)	37
Response button use for VT/VF	12 (1.5%)	24
WCD indications		
Non-ischemic cardiomyopathy (*n* = 249)	6 (2.4%)	15
Heart failure hospitalization (*n* = 230)	3 (1.3%)	4
Acute myocardial infarction (*n* = 128)	6 (4.6%)	10
Post PCI/cardiac surgery (CABG) (*n* = 72)	3 (4.2%)	4
Other risk assessment (*n* = 102)	3 (2.9%)	4
Supraventricular tachyarrhythmias	18 (2.3%)	46
Atrial fibrillation/atrial flutter	12 (1.5%)	38
Supraventricular tachycardia	6 (0.8%)	8
Response button use for SVT	10 (1.3%)	23
Bradycardia/asystole	1 (0.1%)	1

*CABG* Coronary artery bypass grafting, *ICD* Implantable cardioverter-defibrillator, *PCI* Percutaneous coronary intervention, *SVT* supraventricular tachyarrhythmia, *VF* ventricular fibrillation, *VT* ventricular tachycardia, *WCD* wearable cardioverter-defibrillator

#### Phase II: Follow-up period

779 patients finished WCD wearing and entered the Phase 2 of the registry with a planned follow-up time of 12 months. During follow-up, two patients withdrew their permission to be followed further and seven patients were lost to follow-up (Fig. 1).

After WCD prescription, ICD implantation was performed in 289 of 779 patients (37%); whereas in 472 patients (61%), ICDs were not implanted. In 18 patients (2%), status of ICD implantation is unknown (Table 3). Of the 399 patients with LVEF ≤ 35% after WCD prescription, 251 (63%) received ICDs, but 140 (35%) remained without defibrillators; in eight patients (2%), ICD implantation is unknown. Of 380 patients with LVEF > 35%, after WCD prescription, 38 (13%) received ICDs (Table 4). The majority of those (68.4%) received ICDs due to frequent non-sustained VTs recorded during WCD prescription without shock delivery. In 12 patients, the ICD was implanted after further diagnostic work-up during WCD prescription. Five patients (13.2%) were finally diagnosed with an inherited arrhythmia syndrome and in seven patients (18.4%), the ICD was implanted following arrhythmogenic syncope due to ventricular tachyarrhythmias. ICD infection and complications occurred in seven patients, requiring removal of the device in five. During follow-up, 12 out of 289 ICD patients (4%) received appropriate ICD therapies for VT/VF events.

**Table 3:** ICD implantation after WCD wearing by WCD indication

WCD indication	Mean LVEF after WCD wearing	ICD implanted	No ICD implanted	Missing data
Non-ischemic cardiomyopathy	36.4 ± 12.8%	90 (36%)	153 (62%)	5 (2%)
Hospitalization for heart failure	34.4% ± 11.4%	79 (34%)	142 (62%)	9 (4%)
AMI	35.1 ± 10.4%	42 (33%)	84 (66%)	2 (1%)
PCI/CABG	33.9 ± 10.4%	34 (47%)	37 (51%)	1 (2%)
Other risk assessment	44.1 ± 13.7%	44 (43%)	56 (56%)	1 (1%)
Total (*n* = 779)	36.3 ± 12.3%	289 (37%)	472 (61%)	18 (2%)

*LVEF* left ventricular ejection fraction, *WCD* wearable cardioverter-defibrillator, *PCI* percutaneous coronary intervention, *CABG* coronary artery bypass grafting, *ICD* implantable cardioverter-defibrillator, *AMI* acute myocardial infarction

**Table 4:** ICD implantation according to LVEF after WCD wearing

	LVEF ≤ 35%	LVEF > 35%
ICD implantation	251 (63%)	38 (10%)
No ICD implantation	140 (35%)	332 (87%)
ICD status unknown	8 (2%)	10 (3%)
Total (*n* = 779)	399 (51%)	380 (49%)

*ICD* implantable cardioverter-defibrillator, *LVEF* left ventricular ejection fraction, *WCD* wearable cardioverter-defibrillator

**Fig. 2:**
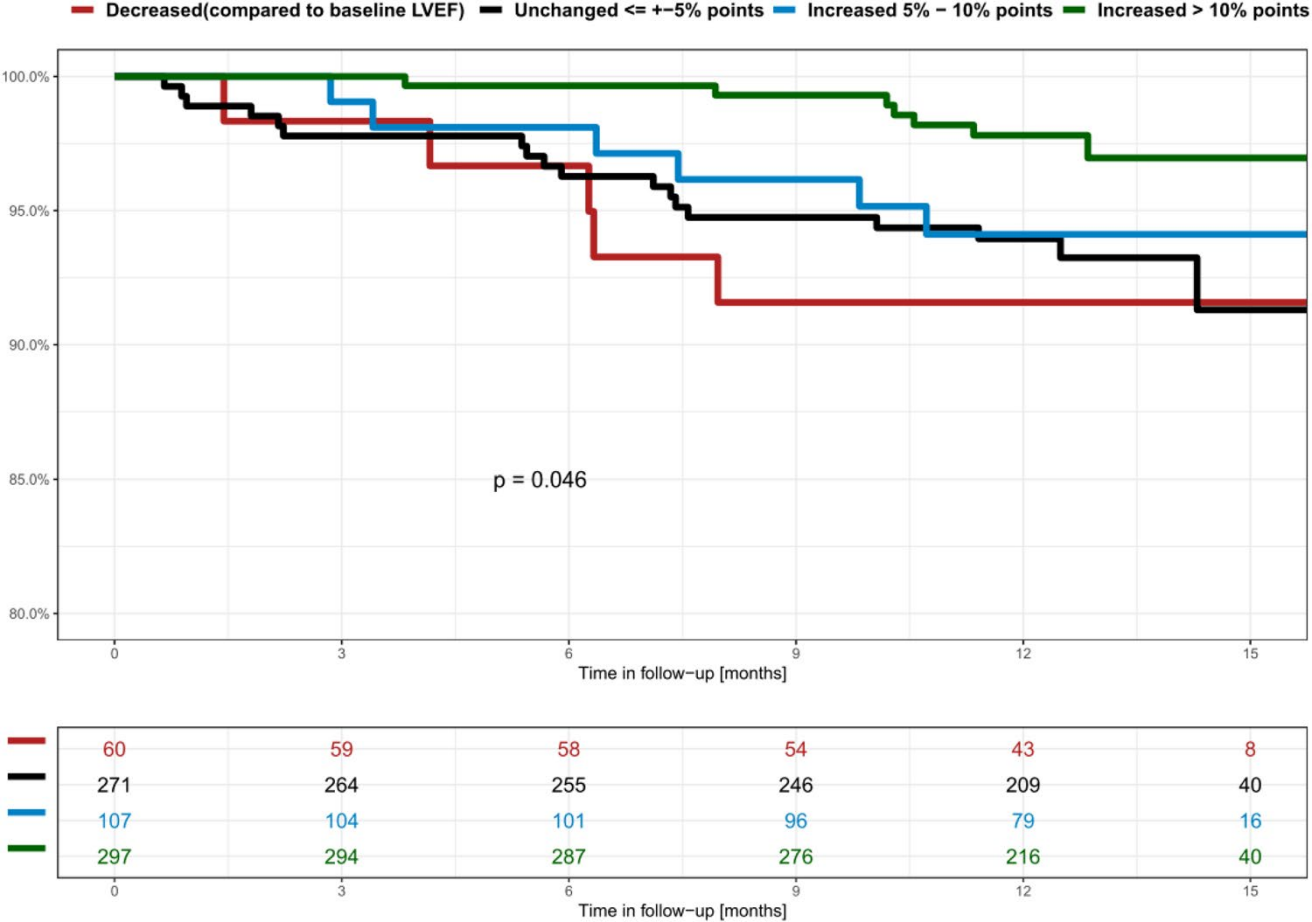
Cumulative survival according to LVEF development during WCD prescription

During phase II follow-up, 40 of 770 patients (5.1%) died. The mode of death was classified as sudden cardiac death in three patients (0.4%); 15 patients (1.9%) died of terminal heart failure; 15 patients (1.9%) of non-cardiac death; and in seven patients (0.9%), the cause of death remained unknown (Table 5). In 27 deaths (67%), LVEF at the end of WCD prescription had either decreased or remained unchanged compared to baseline LVEF. In 29 deceased patients (72%) LVEF after WCD prescription was ≤ 35%. Cumulative survival during the time of follow-up was significantly better for patients in whom LVEF increased > 10% points compared to those with decreased, unchanged or even only slightly increased LVEF after WCD prescription (Fig. 2).

**Table 5:** Mode of death during follow-up after WCD wearing (phase 2)

WCD Indication/deaths	Sudden cardiac death	Heart failure death	Non-cardiac death	Death of unknown cause
Non-ischemic cardiomyopathy (*n* = 7)	1	3	1	2
Heart failure hospitalization (*n* = 9)	1	5	1	2
AMI (*n* = 11)	0	2	7	2
PCI/CABG (*n* = 8)	1	3	3	1
Other risk assessment (*n* = 5)	0	2	3	0
Total (*n* = 40)	3 (7%)	15 (38%)	15 (38%)	7 (17%)

*WCD* wearable cardioverter-defibrillator, *PCI* percutaneous coronary intervention, *CABG* coronary artery bypass grafting, *ICD* implantable cardioverter-defibrillator, *AMI* acute myocardial infarction

**Fig. 3:**
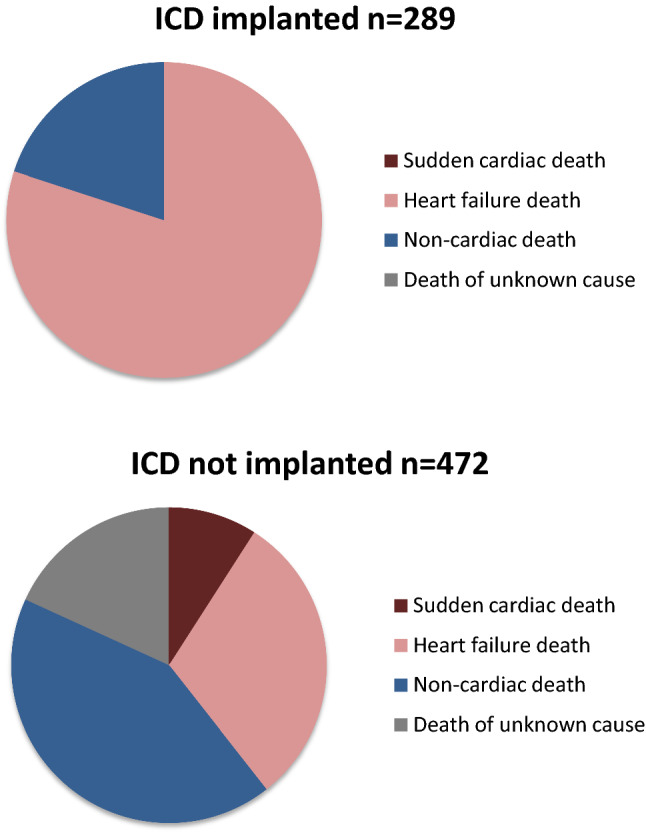
Mode of death according to ICD status

**Fig. 4:**
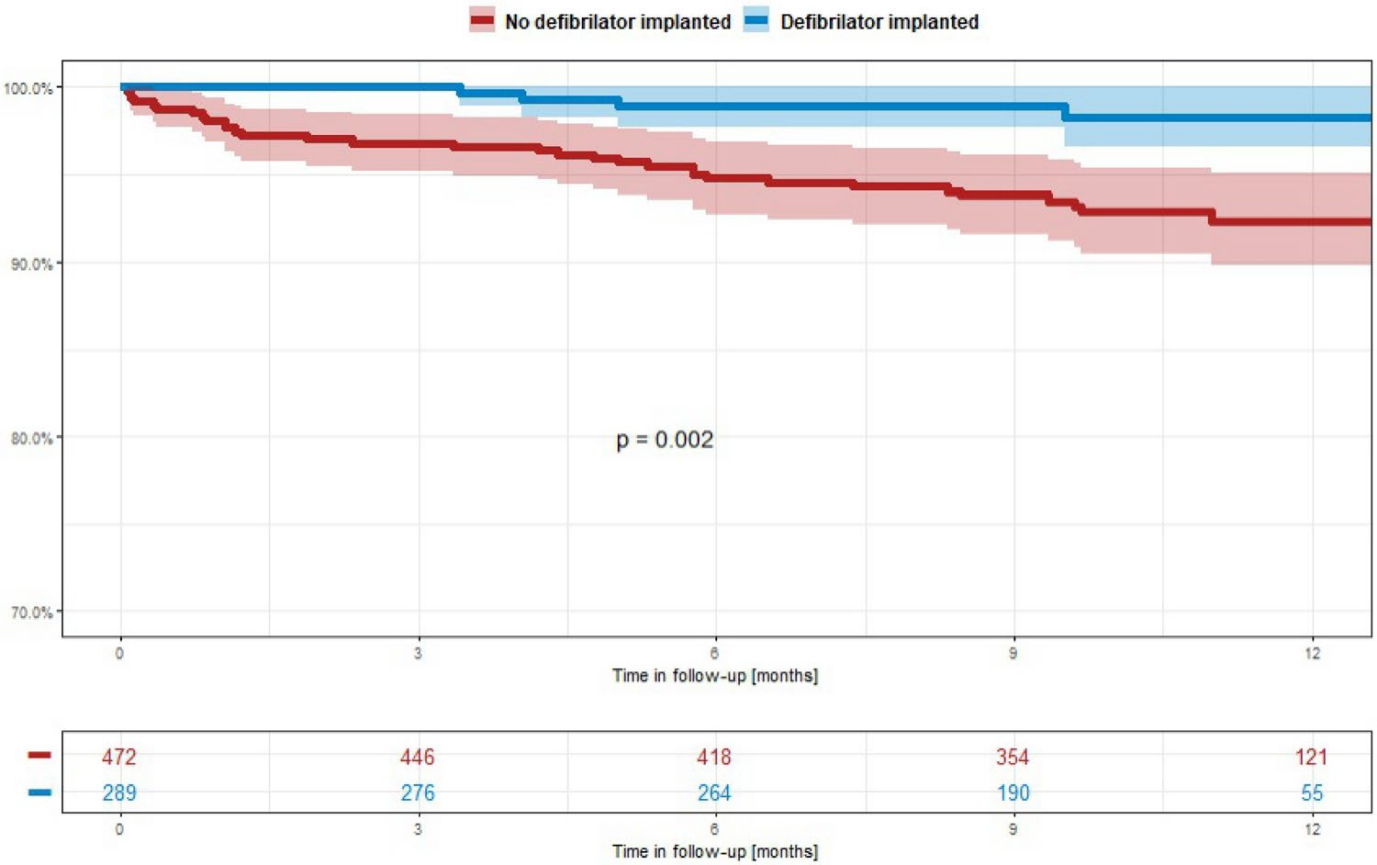
Cumulative survival according to ICD implantation following WCD prescription

Of the 472 patients who did not receive ICDs after WCD prescription, 33 (7%) patients died during follow-up, ten due to heart failure, 14 of non-cardiac death, and three had sudden death. In contrast, of 289 patients with implanted ICDs, five patients (1.7%) died, four due to heart failure and one patient due to non-cardiac death (Fig. 3). Cumulative survival with implanted ICDs after WCD prescription was significantly better compared to patients who were left without ICD therapy (*p* = 0.002) (Fig. 4).

### Development of left ventricular ejection fraction

**Fig. 5:**
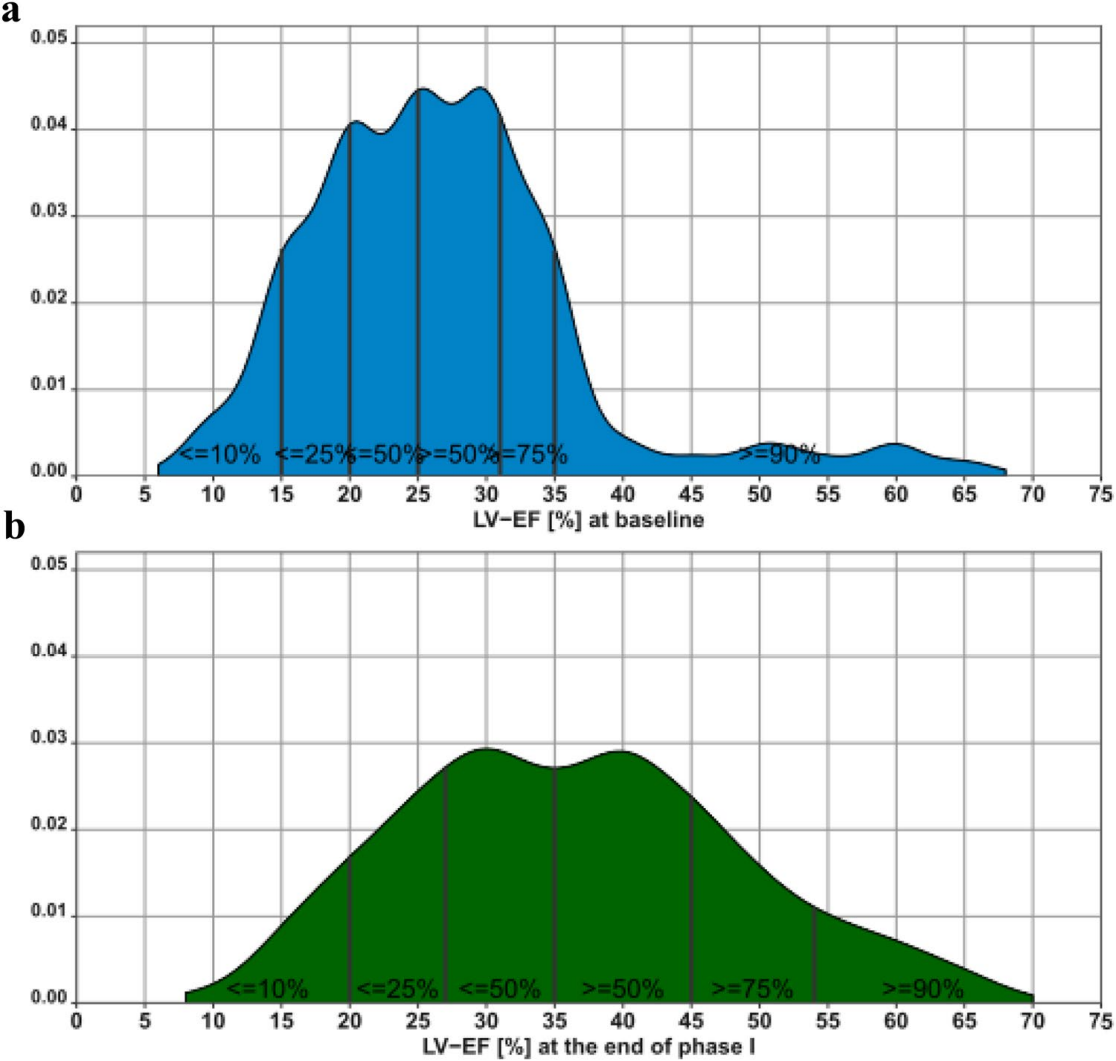
Distribution of LVEF at baseline (**a**) and at the end of Phase 1 (**b**) after initiation and optimization of guideline-directed heart failure medication during WCD prescription

Progression of LVEF following WCD prescription up to 12 months of follow-up was one of the primary objectives of WEARIT-II-EUROPE. At baseline, mean LVEF of 781 patients was 26.9% ± 10.3% and increased to 36.3% ± 12.3% after the end of WCD prescription (*p* < 0.01). LVEF significantly increased in all five WCD indication groups (Table 6). After the end of WCD prescription, 44% of the cohort with an initial LVEF < 35% showed increased LVEF to > 35% (Fig. 5). Until 12-month follow-up, LVEF further increased to a mean of 39.4% ± 12.8%. After WCD prescription LVEF showed further significant increase in patients categorized in “non-ischemic cardiomyopathy”, “acute myocardial infarction” and “heart failure hospitalization”.

**Table 6:** Development of left ventricular ejection fraction baseline, after phase 1 and after phase 2

WCD indication	LVEF baseline (%)	LVEF after phase 1 (%)	*p* value (baseline vs. phase 1)	LVEF after phase 2 (%)	*p* value (phase 1 vs. phase 2)	*p* value (baseline vs. phase 2)
Non-ischemic cardiomyopathy	26.1 ± 10.4	36.4 ± 12.8	< 0.01	40.7 ± 12.4	< 0.01	< 0.01
Heart failure hospitalization	23.6 ± 7.5	34.4 ± 11.4	< 0.01	38.1 ± 13.7	< 0.01	< 0.01
Acute myocardial infarction	26.7 ± 6.6	35.1 ± 10.4	< 0.01	37.6 ± 11.4	< 0.01	< 0.01
Revascularization PCI/CABG	25.7 ± 5.9	33.9 ± 10.4	< 0.01	35.8 ± 11.2	ns	< 0.01
Other risk stratification	38.3 ± 14.3	44.1 ± 13.7	< 0.01	44.1 ± 13.4	ns	< 0.01
Total	26.9 ± 10.3	36.3 ± 12.3	< 0.01	39.4 ± 12.8	< 0.01	< 0.01

*CABG* coronary artery bypass grafting, *LVEF* left ventricular ejection fraction, *PCI* percutaneous coronary intervention, *WCD* wearable cardioverter-defibrillator

## Discussion

The WEARIT-II-EUROPE registry is the first large prospective registry not only analyzing the period of WCD prescription but also providing a 12-month follow-up after WCD prescription. Thus, the focus of WEARIT-II-EUROPE was not the period of WCD prescription but rather the following time with respect to improvement of LVEF and overall mortality outcome.

The main findings of WEARIT-II-EUROPE are: (1) the WCD is protective during risk assessment for ICD indication. All life-threatening ventricular arrhythmic events were effectively terminated by WCD shocks (2) wearing compliance is high. (3) After newly diagnosed heart failure, LVEF improves significantly during WCD prescription and up to 12 months thereafter. (4) Primary preventive ICD implantation was withheld in 35% of patients, despite severely reduced LVEF. (5) Heart failure death and non-cardiac death were the main causes of death at 12-month follow-up.

### WCD period

WCD prescription was an inclusion criterion of WEARIT-II-EUROPE. In contrast to WEARIT US and other WCD registries, the main objective of WEARIT-II-EUROPE was not just the assessment of potential ventricular arrhythmias. The WCD was particularly prescribed to provide a protected period for optimization of GDMT and better risk assessment for potential future ICD implantation.

In WEARIT-II-EUROPE, wear-time compliance with more than 20 h per day was consistent with previous published registries [9–11]. The incidence of ventricular tachyarrhythmia events within the different patient cohorts was comparable with other studies and varied from 1.3 to 4.7% within a mean of 75 ± 47.7 days WCD wearing [10, 12, 13]. The majority of VT/VF events occurred in heart failure with ischemic etiology. The VT/VF event rate confirms the results of the prospective VEST-trial and other WCD registries [11, 14]. It is important to observe that only ten out of 21 VT/VF events needed termination by WCD shocks. In 52% of these events, WCD shocks were withheld by use of the response button. This indicates that half of the VT/VF events were hemodynamically stable, tolerated by the patient and terminated spontaneously within minutes. In WEARIT-II-EUROPE, no ineffective shock delivery occurred, and the incidence of inappropriate shocks (0.3%) was tolerably low. Previously unknown supraventricular tachyarrhythmias were detected in 2.3% of the patients. All in all, the WCD effectively protected patients from sudden arrhythmic death and detected clinically significant new arrhythmias in a total of 3.7% of the patients.

### Development of left ventricular function

During a mean WCD prescription of 75 days allowing optimization of GDMT, mean LVEF significantly increased in all five WCD indication categories (Table 6). Overall, in 44% of all patients, LVEF was higher than 35% at the end of WCD prescription, making ICD implantation redundant or at least not guideline indicated. Of the 51% of patients with a baseline LVEF ≤ 25%, only 22% continued to have a LVEF < 35%.

Time and adequate dosage of medication are the main factors for LVEF recovery [11, 15–17]. Especially in patients with “non-ischemic cardiomyopathy”, “hospitalization for newly diagnosed acute heart failure” and “acute myocardial infarction”, waiting time plays a major role for LVEF recovery. It is important to note that in these WCD categories, further significant increase of LVEF up to 12 months was observed compared to end of WCD prescription. These findings indicate that reverse remodeling needs time and is not completed within the first 2–3 months after heart failure diagnosis. Obviously, more time is needed to evaluate the effect of GDMT on reverse remodeling and heart failure symptoms [18]. Re-assessment of LVEF after the provided time of therapy adjustment is of imminent importance for the appraisal of patients’ overall outcome and for confirmation or deferring of ICD implantation [19–21].

WEARIT-II-EUROPE demonstrates that patients with decreased or unchanged LVEF after WCD prescription have a significantly lower 12-month survival than patients showing more than 10% increase of their LVEF (Fig. 3). Other studies have dearly shown that awaiting LVEF recovery is of great importance [22]. The PREDICTS study assessed independent clinical parameters that predicted significant LVEF recovery to greater than 35% after 3 months in patients after acute myocardial infarction with initial LVEF ≤ 35% [23]. The authors showed that their risk score model identified LVEF recovery beyond 35% in 57% of their patient cohort. Another study of a large patient cohort showed that patients with heart failure and recovery from preexisting reduced LVEF had a lower overall mortality and less frequent hospitalizations than patients with higher but stable preserved LVEF [24]. This indicates that recovery of initially reduced LVEF may be more advantageous and is a better predictor for a favorable overall outcome compared to initially higher but unchanging LVEF [25]. An earlier study in patients with non-ischemic cardiomyopathy found better overall outcome with recovered LVEF and appropriate heart failure therapy compared to those with preexisting higher LVEF and less heart failure symptoms [26]. Our findings confirm results from the PROLONG study indicating that waiting beyond 3 months after heart failure diagnosis may have a beneficial impact on ICD implantation decisions due to continued reverse remodeling of LV function [11].

### Mortality and prognosis following WCD period

WEARIT-II-EUROPE is the first registry providing prospective data on mortality after WCD prescription in a large patient population including assessment of the mode of death with and without ICD implantation.

At the end of WCD prescription, a total of 51% showed a LVEF ≤ 35%. Physicians rejected ICD implantation in 140 patients, although LVEF was measured below 35% after WCD prescription. This demonstrates that the decision for ICD implantation was not solely depending on measurement of LVEF progression but was guided by the general status of patients’ disease process or their expected overall survival. Patients who remained without ICDs showed significantly higher overall mortality (7%) after 1 year compared to patients in whom ICDs were implanted (1.7%). However, the cause of death in patients without ICDs was unrelated to arrhythmic events in at least 73% of all patients. These patients died due to heart failure progression or non-cardiovascular cause, an ICD cannot prevent. In ICD receivers all deaths occurred within 9 months post implantation due to heart failure progression or non-cardiovascular causes. Thus, the results of WEARIT-II-EUROPE strongly support the requirement of individual risk assessment beyond LVEF measurement prior to ICD implantation with the option to reject defibrillator therapy if patients’ condition, co-morbidity and general condition vote against beneficial ICD therapy [27].

Overall mortality after WCD prescription in WEARIT-II-EUROPE was 5.2%. One-year mortality differed between etiologies of heart failure. Worst prognosis was observed in patients with an ischemic cardiomyopathy (acute MI and post-revascularization with severely reduced left ventricular function). Overall mortality in this population was 9.5%. Patients with non-ischemic cardiomyopathy had the best prognosis (1-year mortality 3.6%). Heart failure death and non-cardiac causes were the dominant modes of death. In WEARIT-II US, 1-year mortality in patients with ischemic etiology was only 3% (with implanted ICDs) and 4% (without ICDs). Mortality in non-ischemic cardiomyopathy (3%) was similar in the two WEARIT registries [28]. Mode of death during follow-up was not assessed in WEARIT-II-US registry. Thus, differences of mortality between Europe and the USA cannot be sufficiently explained.

Two important trials have shown that early ICD implantation after acute myocardial infarction did not provide long-term overall benefit although arrhythmic mortality was significantly reduced [29, 30].

One may speculate whether outcomes of the two trials would have been different if patient randomization would have started after a 2- or 3-month WCD prescription period. Early post-infarction sudden deaths most likely would have been avoided, fewer patients would have received ICDs because of subsequently no longer indicated ICD therapy, and later on, a true benefit of ICD therapy after myocardial infarction with persistent low LVEF could have been elaborated. In addition, the early unpredictable non-arrhythmic deaths that may have influenced overall outcomes of the trials could have been censored.

Results of these two trials do not contradict the use of the WCD after acute myocardial infarction. They rather request risk assessment prior to ICD implantation balancing the risk of arrhythmic vs. non-arrhythmic death [31]. The results of the DANISH trial stress the ongoing discussion on the true benefit of primary prevention ICD therapy in patients with NICM [32]. Sudden death was significantly reduced in the ICD group without positive impact on overall mortality. Mode of death in the ICD group was shifted. DANISH supports the need for thorough individualized risk evaluation in patients with various forms of NICM [32, 33]. Patients with NICM may have an increased risk for ventricular tachyarrhythmia, particularly in the early phase with acute heart failure [34–36]. Thus, risk assessment under protection of a WCD can be considered.

The recently published VEST trial [14] randomized patients with a low LVEF after acute myocardial infarction to a WCD arm or no WCD arm. After 3 months, no difference in arrhythmic deaths between the two groups was found, but patients in the WCD group had a significantly lower overall mortality. Unfortunately, the VEST trial ended after WCD prescription. So, the impact of WCD prescription on long-term follow-up remains unclear. The results of the VEST trial are not comparable to our registry. They cannot be applied to negate the benefit of early post-infarction protection against sudden arrhythmic death. WCD compliance in the VEST trial was very poor (only 14 h per day). In a real-world setting, this is unacceptable, as the WCD can only terminate VT/VF if properly and permanently worn. This low WCD compliance is responsible that only nine out of 25 patients experiencing sudden death have worn the WCD at the time of death. The importance of wear compliance was stressed in the recently published as-treated analysis of VEST [37]. In addition, the WEARIT-II-EUROPE registry is hard to compare with VEST because VEST tried to test the WCD as a “therapeutic tool” but not as an approach for protected risk stratification after myocardial infarction.

Current guidelines and consensus statements have listed prescription of the WCD as a Class IIa or IIb with level of evidence C [1, 38]. However, beyond the proven efficacy of defibrillation, WEARIT-II-EUROPE showed that the WCD represents a promising approach for protected individual risk assessment prior to deciding for ICD implantation in patients with a presumed but not yet confirmed risk of sudden cardiac death. Appraisal of patients’ risks of non-arrhythmic death or overall mortality regarding long-term benefit of defibrillator therapy is the future challenge of sudden cardiac death primary prevention. The results of the WEARIT-II-EUROPE registry support this statement and strengthen the request to perform individualized risk assessment prior to ICD implantation.

## Limitations

WEARIT-II-EUROPE represents the first prospective registry with pre-specified categorized WCD prescriptions testing the value of risk assessment under the protecting umbrella of the WCD. The registry protocol did not request specific enrollment criteria or had defined exclusion criteria for WCD prescription. Treatment, diagnostics and procedures during and after WCD prescription were left to physicians’ discretion and were not mandated by the registry protocol. Thus, the registry represents a real-world scenario. Presumably, follow-up beyond 1 year would have provided additional information on patients’ long-term outcome. However, this would have exceeded our main purpose to analyze LVEF development and mortality after WCD prescription. Our registry did not aim to evaluate the long-term outcome of primary prevention ICD therapy for various underlying diseases. Only 1% of enrolled patients was lost during follow-up or withdrew from the registry. In seven of the 40 patients, the precise cause of death remained undetermined, information on ICD implantation could not be recruited in 2% of all enrolled patients; however, no death event remained unrecognized. Cost–benefit analysis was not part of the WEARIT-II-EUROPE registry study protocol. However, a recently published cost-effectiveness study of using the WCD early after acute myocardial infarction has demonstrated the positive cost-effectiveness of the WCD and concludes that the WCD is economically attractive when compared with other generally accepted treatments [39].

## Conclusions

In WEARIT-II-EUROPE, the WCD was prescribed for individualized risk assessment prior to potentially indicated ICD implantation in patients with an early, but possibly not permanent risk of sudden arrhythmic death. During the time of protected risk assessment, information on improvement of left ventricular function and response to medical therapy can be gathered and facilitates estimation of patients’ overall prognosis. Using the WCD for individualized risk assessment is a promising approach to avoid unnecessary ICD implantation and may help increasing the benefit of primary preventive ICD therapy.
