# Correction to: Protected risk stratification with the wearable cardioverter-defibrillator: results from the WEARIT-II-EUROPE registry

**DOI:** 10.1007/s00392-020-01677-y

**Published:** 2020-06-24

**Authors:** Christian Veltmann, Stefan Winter, David Duncker, Carsten G. Jungbauer, Nadine K. Wäßnig, J. Christoph Geller, Julia W. Erath, Olaf Goeing, Christian Perings, Michael Ulbrich, Mattias Roser, Daniela Husser, Laura S. Gansera, Korkut Soezener, Frank Michael Malur, Michael Block, Thomas Fetsch, Valentina Kutyifa, Helmut U. Klein

**Affiliations:** 1 Rhythmology and Electrophysiology, Department of Cardiology and Angiology, Hannover Medical School, Carl-Neuberg-Str. 1, 30625 Hannover, Germany; 2 St. Vinzenz-Hospital Köln, Cologne, Germany; 3 University Hospital Regensburg, Regensburg, Germany; 4 Universitätsklinikum Dresden, Herzzentrum, Dresden, Germany; 5 Arrhythmia Section, Division of Cardiology, Zentralklinik Bad Berka, Bad Berka, Germany; 6 Otto-Von-Guericke University School of Medicine, Magdeburg, Germany; 7 Abteilung für Klinische Elektrophysiologie, Medizinische Klinik III, Universitätsklinikum Frankfurt, Frankfurt, Germany; 8 Sana Klinikum Lichtenberg, Berlin, Germany; 9 St.-Marien Hospital Lünen, Lünen, Germany; 10 Klinikum Siegburg, Siegburg, Germany; 11 Klinikum Benjamin Franklin, Charité Berlin, Berlin, Germany; 12 Klinik für Kardiologie, Herzzentrum Leipzig, Leipzig, Germany; 13 Klinik für Kardiologie, Klinikum Augsburg, Augsburg, Germany; 14 Klinikum Frankfurt-Hoechst, Frankfurt, Germany; 15 Helios Klinikum Erfurt, Erfurt, Germany; 16 Klinik für Kardiologie, Klinikum Augustinum München, Munich, Germany; 17 CRI-Clinical Research Institute München, Munich, Germany; 18 Medical Center, Clinical Cardiovascular Research Center, University of Rochester, Rochester, NY USA

## Correction to: Clinical Research in Cardiology 10.1007/s00392-020-01657-2

The original version of this article unfortunately contained mistakes.

Carsten G. Jungbauer was missing in the author line and the spelling of the name Thomas Fetsch was incorrect.

The original article has been corrected.

